# Transfer Printing and its Applications in Flexible Electronic Devices

**DOI:** 10.3390/nano9020283

**Published:** 2019-02-18

**Authors:** Honglei Zhou, Weiyang Qin, Qingmin Yu, Huanyu Cheng, Xudong Yu, Huaping Wu

**Affiliations:** 1 Department of Engineering Mechanics, School of Mechanics, Civil Engineering and Architecture, Northwestern Polytechnical University, Xi’an 710129, China; hongleizhou@mail.nwpu.edu.cn (H.Z.); mengg@nwpu.edu.cn (W.Q.); yuxudong@mail.nwpu.edu.cn (X.Y.); 2 State Key Laboratory for Strength and Vibration of Mechanical Structures, Xi’an Jiaotong University, Xi’an 710049, China; 3 Department of Engineering Science and Mechanics, The Pennsylvania State University, University Park, PA 16802, USA; 4 Key Laboratory of Special Purpose Equipment and Advanced Manufacturing Technology, Zhejiang University of Technology, Ministry of Education and Zhejiang Province, Hangzhou 310014, China; wuhuaping@gmail.com

**Keywords:** transfer printing, flexible electronic devices, interfacial adhesion

## Abstract

Flexible electronic systems have received increasing attention in the past few decades because of their wide-ranging applications that include the flexible display, eyelike digital camera, skin electronics, and intelligent surgical gloves, among many other health monitoring devices. As one of the most widely used technologies to integrate rigid functional devices with elastomeric substrates for the manufacturing of flexible electronic devices, transfer printing technology has been extensively studied. Though primarily relying on reversible interfacial adhesion, a variety of advanced transfer printing methods have been proposed and demonstrated. In this review, we first summarize the characteristics of a few representative methods of transfer printing. Next, we will introduce successful demonstrations of each method in flexible electronic devices. Moreover, the potential challenges and future development opportunities for transfer printing will then be briefly discussed.

## 1. Introduction

The increasing popularity of intelligent terminals has driven the rapid development of bio-integrated and implantable electronic devices. These classes of emerging high-performance electronics are lightweight, flexible, and highly sensitive, which opens application opportunities in skin-like electronics, wearable health monitoring devices, and human-machine interfaces. A few representative examples used in biomedical diagnostics include flexible digital x-ray detectors, medical balloon catheters, bio-integrated neural electrode arrays, and soft surgical gloves. Because of the intrinsically flexible and stretchable properties, low-cost fabrication processes, and compatibility with the solution process, the use of organic materials for flexible and stretchable electronics is straightforward [1,2,3,4,5]. However, compromised electrical properties limit their use in certain applications where high performance is desirable [5,6,7]. As an alternative to intrinsically stretchable organic materials, inorganic semiconducting and conducting materials designed in a stretchable layout exhibit outstanding electrical properties, such as high conductivity and charge carrier mobility, yielding devices with performances comparable to those fabricated with conventional wafer-based technologies [6,7,8].

As the high-performance of a device hinges on its fabrication process, it is highly desirable to fabricate stretchable inorganic devices on the conventional substrate with the photolithographic or other similar processes [9,10,11]. To provide a robust handling substrate, and also an interface to the soft tissues, the fabricated inorganic devices would need to be integrated onto the flexible and stretchable substrates [12,13,14,15,16,17,18]. The process to transfer the devices fabricated from the growth (donor) substrate to the receiving substrate can be achieved via a stamp by a technique termed as transfer printing [19,20,21,22,23,24].

**Figure 1:**
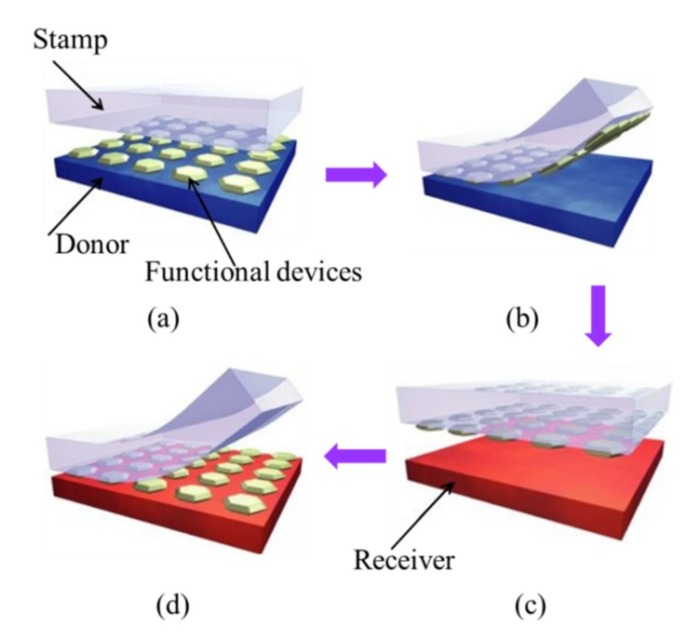
Schematic illustration of the transfer printing process: (**a**) Laminating the stamp against the donor substrate, on which the functional devices are fabricated; (**b**) peeling the stamp to transfer the functional devices from the donor substrate onto the stamp; (**c**) bringing the inked stamp into contact with the receiving substrate (receiver); (**d**) peeling the stamp to print the functional devices onto the receiver. Reprinted with permission from [24]; Copyright 2006, Nature Publishing Group.

As one of the most widely used methods for the fabrication of flexible and stretchable inorganic photonic/electronic devices, transfer printing [22,25,26,27,28,29] provides a versatile route to deterministically assemble micro- and nano-materials into spatially organized, functional arrangements with two- and three-dimensional layouts [30,31,32]. The transfer printing method is also suitable for large-scale assembly [7,19,33,34,35,36,37,38,39]. The typical transfer printing process consists of two steps: Pickup and printing (Figure 1). In the pickup step, functional device components (e.g., micro-/nano-membranes, ribbons, nanowires, nanotube, etc.) pre-fabricated on the donor substrate are first picked up onto a stamp. In the printing step, the inked stamp is then brought into contact with the receiver, followed by the removal of the stamp to print the device components onto the receiving substrate (receiver). The stamp used in both the pickup and printing steps is clearly the key to the successful implementation of the transfer printing process with a high fidelity and easy control [25,40,41].

**Figure 2:**
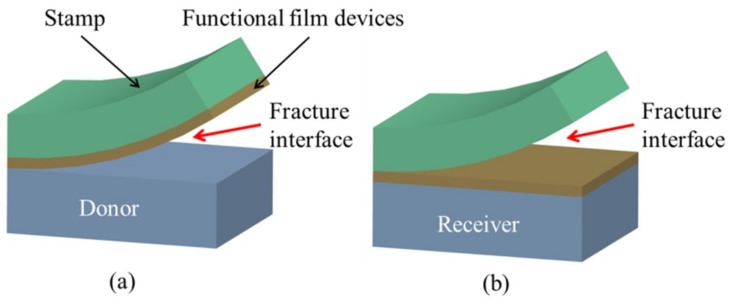
The diagram to show the (**a**) pickup and (**b**) printing steps in the transfer printing process. In the pickup step, the crack propagates at the device/donor interface, whereas delamination occurs at the device/stamp interface in the printing step. Reproduced with permission from [25]; Copyright 2007, American Chemical Society.

The transfer printing process involves the competing fracture at the stamp/device interface and the device/substrate (donor or receiver) interface. The fracture at the stamp/device interface occurs in the printing step, whereas the fracture at the device/donor interface occurs in the pickup step [24,25,42]. The successful pickup requires the adhesion strength at the stamp/device interface to be larger than that at the device/donor interface, resulting in delamination at the device/donor interface to transfer the functional devices onto the elastomeric stamp (Figure 2a). Conversely, the adhesion strength at the device/receiver interface is stronger than that at the stamp/device interface in the printing step. Consequently, peeling off the stamp prints the functional devices onto the receiver (Figure 2b).

As the successful transfer printing process hinges on the modulation of the interfacial adhesion at different material/structural interfaces [40], efforts have been dedicated to the exploration of feasible methods for the modulation of interfacial adhesion strength. Among the various techniques being developed, the straightforward ones include kinetic adhesion control [24,25,43,44], modification of the surface chemistry [45,46], and solvent-assisted adhesion control [47,48]. Though effective, these methods typically involve the use of the solvent or etchant to remove sacrificial layers, which would cause irreversible damage to the various functional components in the device system and compromise the device performance. As a result, the techniques that are based on mechanical principles for reversible adhesion modulation have started to gain momentum.

Transfer printing technology is associated with several salient advantages: (1) The device components fabricated with the conventional wafer-based technologies enable high performance of the electronic systems; (2) the deterministic assembly is efficient, with high precision; (3) the transfer printing process is repeatable even at large-scale integration, such as the roll-to-roll application for both 2D and 3D layouts [49]; (4) certain transfer printing techniques can be operated at room temperature with low cost; and (5) it is applicable to a wide range of structures and materials, ranging from the assembly of micro-/nano-structures in various shapes and sizes to materials that include organic molecular materials [50,51]; inorganic semiconducting materials, such as GaAs/GaN/etc. [52]; functional polymers [53]; metals; piezoelectric materials; and many others.

Given the existing reviews on the technique of transfer printing that mainly focus on the mechanics, materials, and applications [30,54,55,56], this review will summarize the recent developments of transfer printing techniques in Section 2 and their applications in the fabrication process for various flexible and stretchable electronic devices in Section 3. The challenges and future directions of transfer printing will then be discussed in Section 4.

## 2. Transfer Printing Methods

To explore the full potential of different transfer printing techniques, it is important to understand the underlying mechanisms of different techniques, as well as the advantages and limitations of each transfer printing method.

### 2.1. Kinetically Controlled Transfer Printing

**Figure 3:**
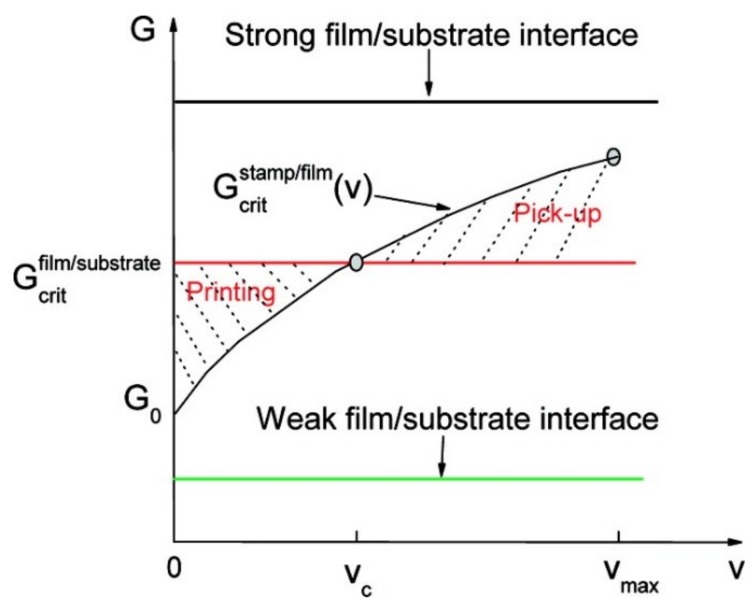
Schematic diagram of critical energy release rates at the film/substrate and film/stamp interfaces as a function of the peeling velocity. In the pickup step, the critical energy release rate at the film/stamp interface is larger than that at the film/substrate interface, leading to the crack propagation at the film/substrate interface and a retrieval of the device film from the donor substrate onto the stamp. In the printing step, the critical energy release rate at the film/stamp interface is smaller than the critical energy release rate at the film/receiver interface, which allows the crack propagation at the film/stamp interface and helps release the device film onto the receiver substrate. Reproduced with permission from [25]; Copyright 2007, American Chemical Society.

Based on the kinetically dependent nature of the viscoelastic stamp [24,25,44], the adhesion strength between the stamp and the functional devices can be modulated by the retraction speed of the stamp. Without using the adhesive layer, van der Waals forces are sufficient in this mode of operation. While the substrate and functional device components (modeled as thin films) are elastic, the critical energy release rate at the film/substrate interface, $G_{crit}^{film/substrate}$, remains unchanged during the transfer printing process. However, with the use of the viscoelastic stamp, the critical energy release rate at the stamp/film interface, $G_{crit}^{stamp/film}$, is rate-sensitive and depends on the peeling velocity. A viscoelastic model, validated by the numerical and experimental results, describes this rate-dependent behavior and the result shows that the pull-off force increases with the peeling velocity [57]. The critical energy release rate, $G_{crit}^{stamp/film}$, that monotonically increases with the peeling velocity, *v*, (Figure 3) can be expressed as:

(1) $$G_{crit}^{stamp/film}(v)=G_{0}\left[1+{\left(\frac{v}{v_{0}}\right)}^{n}\right]$$

where $G_{0}$ is the critical energy release rate when the peeling velocity approaches zero, $v_{0}$ is the reference peeling velocity at which the critical energy release rate equals to $2G_{0}$, and the exponent, *n*, is a scaling parameter that can be obtained by the experiment.

By adjusting the peeling velocity applied on the viscoelastic stamp, the adhesion at the device/stamp interface can be easily modulated to yield controlled delamination: A sufficiently high velocity for pickup (Figure 2a) and then a slow retraction for printing (Figure 2b). Though this kinetically controlled transfer printing technique provides a pragmatic, easy-to-operate tactic to assemble functional devices onto various flexible substrates, the range of modulation is limited due to the restriction of the peeling speed. Moreover, this method is not applicable when the receiver is also viscoelastic, limiting its application to certain classes of materials as the receiver.

### 2.2. Thermal Release Transfer Printing

**Figure 4:**
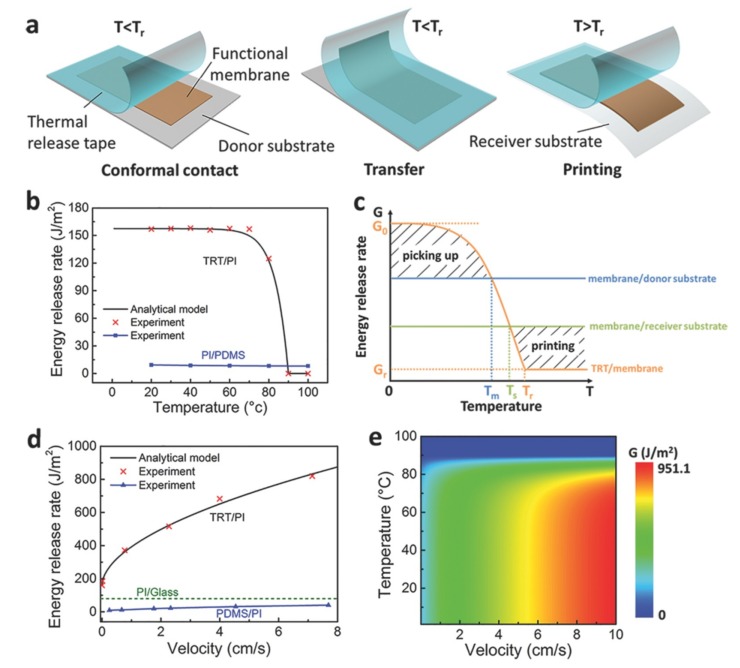
(**a**) Schematic illustration of transfer printing that uses a thermal release tape. The energy release rate at the TRT/Polyimide (PI) and PI/Polydimethylsiloxane (PDMS) interfaces as a function of (**b**) the temperature and (**d**) peeling velocity. (**c**) The modulation of the energy release rate via temperature is used for the pickup and printing steps. (**e**) A contour map shows the energy release rate at the TRT/PI interface as a function of the temperature and peeling velocity. Reproduced with permission from [58]; Copyright 2017, WILEY-VCH Verlag GmbH & Co., KGaA, Weinheim.

As one of the most widely used methods, transfer printing based on the use of a thin and flexible thermal release tape (TRT) is simple to operate and a fracture mechanics model is used in the analysis [58]. The technique relies on the large and irreversible reduction in the adhesion strength when the TRT is heated over the transition temperature (i.e., 100 °C) (Figure 4). The quantitative dependence of the energy release rate at the functional membrane/TRT interface is characterized by Equation (2), which is a function of the temperature and peeling velocity. With the additional control in the temperature, the ratio of strong to weak adhesion becomes larger when compared to the technique that only uses the kinetic control:

(2) $$G_{TRT/membrane}(v,T)=\{\begin{array}{ll} \left[-e^{\gamma \left(T-T_{r}\right)+\ln\left(G_{0}-G_{r}\right)}+G_{0}\right]\left[1+{\left(\frac{v}{v_{0}}\right)}^{n}\right] & T\leq T_{r}\\ G_{0}\left[1+{\left(\frac{v}{v_{0}}\right)}^{n}\right] & T>T_{r} \end{array}$$

where T is the temperature, e is the Euler’s constant, $G_{r}$ is the critical energy release rate when the adhesives on TRT are deactivated, $T_{r}$ is the transition temperature, γ is a material parameter, and the other parameters are the same as in Equation (1).

The modulation of the critical energy release rate at the TRT/membrane interface is mainly controlled by the peeling velocity and temperature as described in Equation (2). The critical energy release rate, $G_{TRT/membrane}$, remains almost unchanged for temperatures below 70 °C (Figure 4b), which is much larger than the critical energy release rate at the membrane/donor interface, $G_{membrane/donor\;substrate}$. Because the crack propagation occurs at the device/substrate interface, the membrane devices could be easily picked up. When the TRT/membrane system is heated above 80 °C, the critical energy release rate, $G_{TRT/membrane}$, significantly decreases to be smaller than the critical energy release rate at the membrane/receiver interface, $G_{membrane/receiver\;substrate}$. Upon peeling off the TRT, the crack propagates at the TRT/membrane interface, which facilitates the printing of the membrane devices onto the receiver substrate. As the critical energy release rate, $G_{TRT/membrane}$, can be modulated with the peeling velocity and temperature over a large range (Figure 4d,e), the thermal release transfer printing represents a powerful method in the device integration.

### 2.3. Water-Assisted Transfer Printing

**Figure 5:**
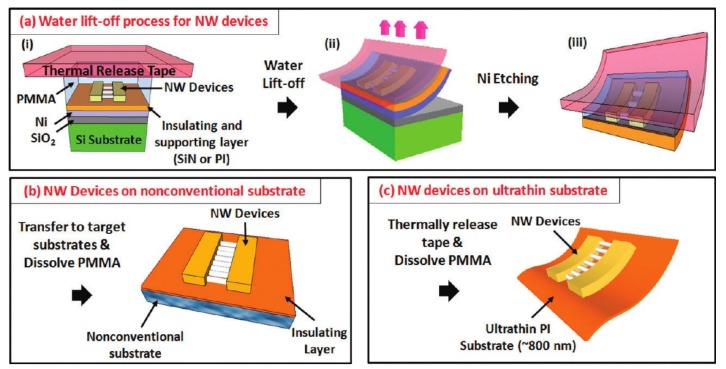
Schematic illustration of water-assisted transfer printing. (**a**) After depositing a thin Ni layer on the Si wafer coated with SiO_2_, the nanowire (NW) devices are fabricated on an insulating and supporting layer (SiN or PI). Lift-off in a water bath at room temperature allows the transfer of the nanowire device and the Ni layer onto the thermal release tape. A subsequent Ni etching removes the Ni layer. The devices with an insulating layer can then be printed onto (**b**) a nonconventional substrate or (**c**) an ultrathin polyimide substrate. Reproduced with permission from [59]; Copyright 2011, ACS publications.

With the use of a thin Ni layer between the device and the donor (e.g., Si wafer coated with SiO_2_), the water-assisted transfer printing method (WTP) is developed to integrate electronic devices, such as those based on nanowires (NW) on different substrates (Figure 5) [59,60,61]. Peeling-off in a water bath at room temperature transfers the device and the Ni layer onto the thermal release tape. Following the Ni etching and a printing step delivers the device to different non-conventional substrates without defects.

The successful operation of WTP clearly depends on the water-assisted interfacial fracture at the Ni/SiO_2_ interface. Deposition of the Ni layer on the Si wafer coated with SiO_2_ forms nickel silicate or nickel oxide, which results in Ni hydroxide when it is immersed in the water. As both the SiO_2_ and Ni hydroxide are hydrophilic, water can quickly penetrate at the interface to result in interface separation. Because the lift-off is operated at room temperature, the possible damage from high temperatures can be avoided. The detailed working principle of the interfacial debonding process is studied through theory, experiment, and finite element analysis [62].

**Figure 6:**
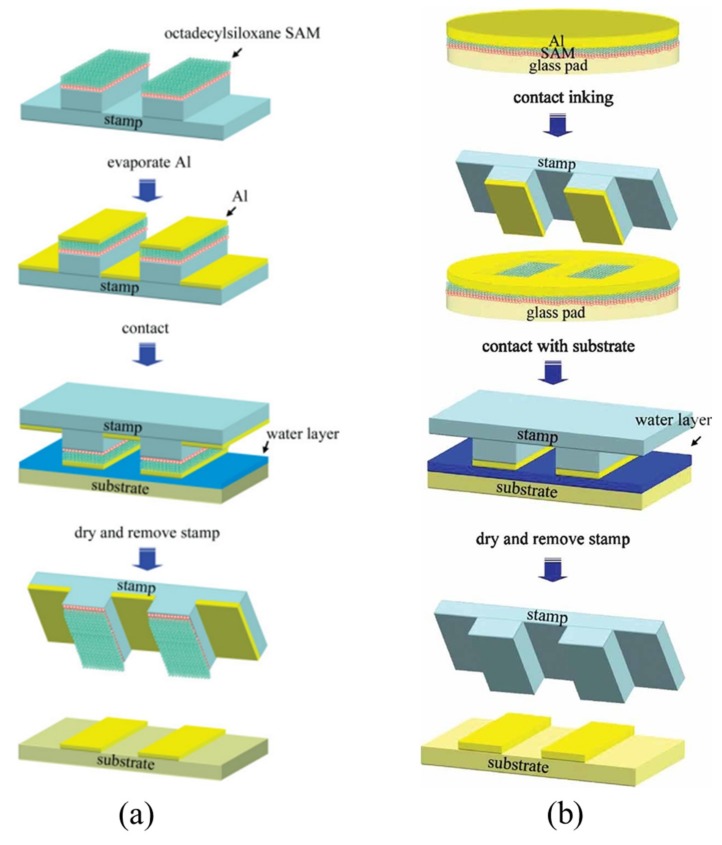
Schematic illustration of the water-mediated transfer printing process: (**a**) Firstly, the patterned PDMS stamp coated by SAM is fabricated. Secondly, the Al thin film is evaporated onto the SAE layer. Then, the inked stamp is brought to contact with the target substrate covered by a thin water layer. Finally, peeling off the stamp from the substrate after drying completes the print process. (Reproduced with permission from [63]; Copyright 2007, WILEY-VCH Verlag GmbH & Co., KGaA, Weinheim). (**b**) The Al thin film is first deposited onto the SAM-coated glass pad (donor substrate), where the SAM layer facilitates the release of the Al thin film onto the PDMS stamp. Then, the inked stamp is brought into contact with the substrate coated with a water layer to enhance the adhesion strength between the Al thin film and the receiver. Finally, the stamp is removed to print the Al film on the receiver upon the drying of the water (Reproduced with permission from [64]; Copyright 2009, WILEY-VCH Verlag GmbH & Co., KGaA, Weinheim).

In a different approach, a water-mediated transfer printing method is developed to print Al thin films on a substrate as shown in Figure 6a [63] and Figure 6b [64]. Although there are some slight differences between the two transfer printing processes, their working principle is the same. That is, the self-assembled monolayers (SAM) layer weakens the adhesion strength between the Al thin film and the stamp/glass inkpad in the pickup step and enables the facile and complete release of the Al thin film. The water layer on the substrate, as the most critical part, serves as an adhesion layer, promoting the adhesive bonding between the Al thin film and the target substrate. For example, as shown in Figure 6b, spontaneously formed on the donor glass substrate, a thin organic self-assembled monolayer (SAM), such as octadecylsiloxane, facilitates the release of Al onto the PDMS stamp in the pickup step. In the printing step, the water layer pre-formed on the receiver surface promotes the interface adhesion at the Al/receiver interface.

### 2.4. Surface Relief Structure-Assisted Transfer Printing

**Figure 7:**
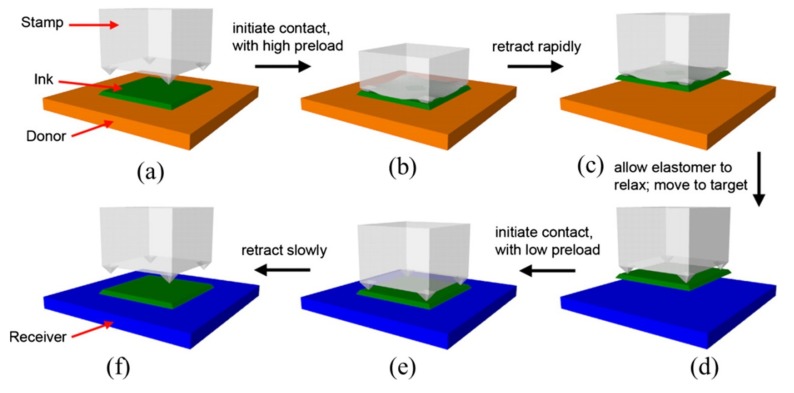
Schematic of surface relief structure-assisted transfer printing. (**a**) A stamp with four pyramidal microtips at each corner is first brought into contact with the functional device components, followed by (**b**) an external pressure to result in the collapse of the microtips. (**c**) Rapid retraction of the stamp transfers the device component onto the stamp. (**d**) The inked stamp is then transported to the receiver. (**e**) Without the pressure, the microtips on the stamp recover to minimize the contact area at the stamp/device interface. (**f**) A slow retraction then prints the device component onto the receiver. Reproduced with permission from [67]; Copyright 2010, National Academy of Sciences of the United State of America.

The interfacial adhesion strength dominated by van der Waals interaction is directly related to the contact area at the interface. Leveraging the idea of structural collapse [65,66], a surface relief structure-assisted transfer printing method was developed to mimic the reversible dry adhesion at the gecko’s foot [67]. Instead of using stamps with a flat surface, the soft stamp is designed with pyramidal microtips on its surface (Figure 7). In the pickup step, the applied pressure collapses the pyramidal microtips to increase the contact area, thereby enhancing the adhesion at the stamp/device interface for pickup. After placing the inked stamp onto the receiver, the removal of the pressure leads to the recovery of the pyramidal microtips for a significantly reduced contact area, minimizing the adhesion strength at the stamp/device interface for printing (Figure 7e). It should be noted that it is also possible to combine the principle of kinetically dependent adhesion by a rapid peeling in the pickup (Figure 7c) and/or a slow retraction in the printing (Figure 7f) to further increase the range of modulation in the interfacial adhesion strength.

As it is desirable to have a reversible adhesion, it is critical to design the geometry of the microtips, such as its height. Two- and three-dimensional mechanics models are established [68] to determine the range of heights. The expressions of the minimum and maximum heights of the microtips are deduced in the two- and three-dimensional models to investigate the influence of the height of the microtips on the reversible adhesion strength. Verified by the experiments, the analytical study provides useful guidelines in the design of surface structures.

**Figure 8:**
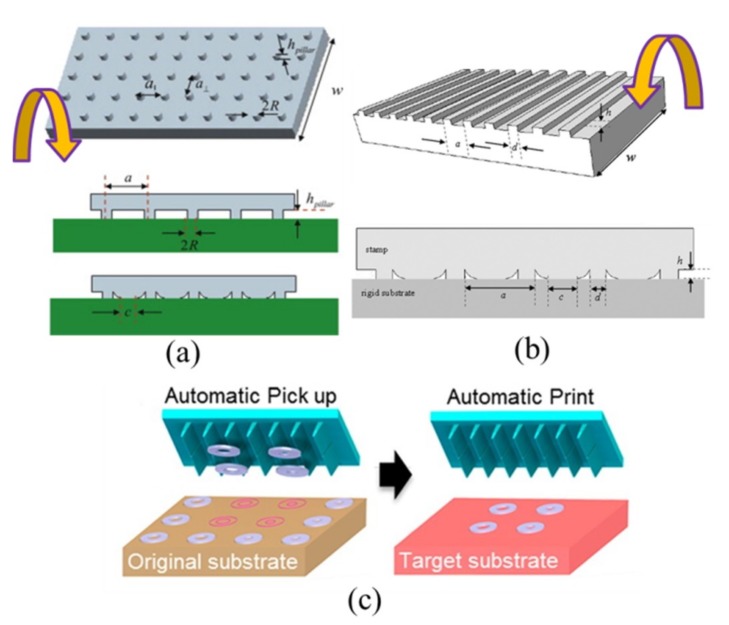
Various viscoelastic stamps with different surface relief structures: (**a**) Micro-pillars (Reproduced with permission from [69]; Copyright 2013, Elsevier), (**b**) grating-like reliefs (Reproduced with permission from [70]; Copyright 2013, AIP Publishing LLC), and (**c**) angled micro-flaps (Reproduced with permission from [72]; Copyright 2014, American Chemical Society).

Similar to the idea of changing the contact area at the stamp/device interface, a variety of viscoelastic stamps with different surface relief structures have been designed, including patterned pillars [69], grating-like reliefs [70,71], angled micro-flaps [72], and many others (Figure 8).

### 2.5. Shear-Assisted Transfer Printing

**Figure 9:**
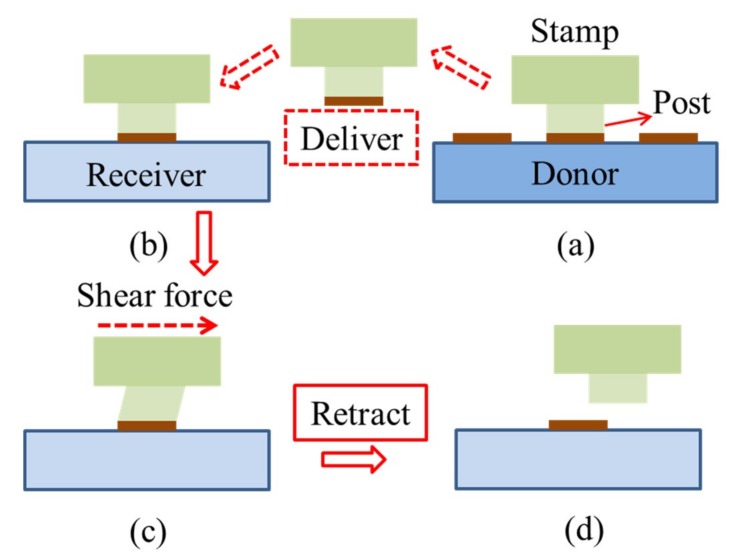
Schematic illustration of shear-assisted transfer printing. (**a**) The stamp is retracted rapidly from the donor substrate to pick up the device component. (**b**) After bringing the inked stamp into contact with the receiver, (**c**) a shear force is applied to reduce the pull-off force at the device/stamp interface. (**d**) A slow retraction then prints the device component onto the receiver. Reproduced with permission from [75]; Copyright 2012; Elsevier.

With the same inspiration from the gecko’s foot, the directionally dependent dry adhesion has also been applied to transfer printing, resulting in the shear-assisted technique [73,74]. The analytical model [75], validated by the experiment, indicates that a shear displacement on the stamp greatly reduces the critical energy release rate at the device/stamp interface, which helps the separation at the interface. The pull-off force needed for delamination at the device/stamp interface decreases almost linearly with the increasing shear strain. In view of this, the shear displacement is applied in the printing step (Figure 9).

### 2.6. Transfer Printing Based on Shape Memory Polymer

**Figure 10:**
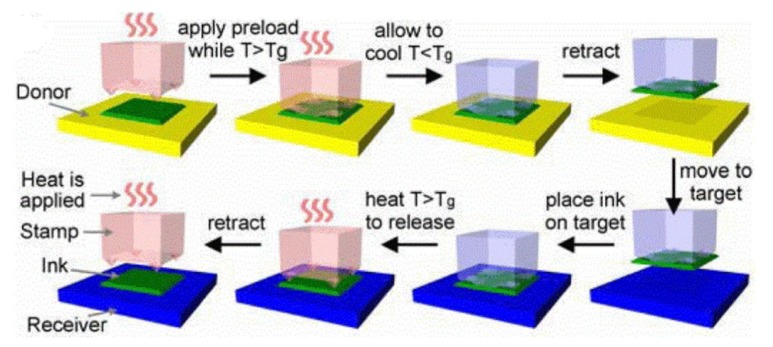
Schematic illustration for the transfer printing method based on shape memory polymers. When the temperature is heated above the transition temperature, applying a preload leads to collapse at the microtips and increases the contact area between the stamp and the donor to yield an increased adhesion at the ink/donor interface. Cooling down the temperature to a value below the transition temperature, the shape memory polymer stamp is programmed to this temporary shape for the transferring of the ink in contact. After bringing the inked stamp into contact with the receiver, heating above the transition temperature allows the SMP stamp to recover its permanent shape with a significantly reduced contact area for a much lower interfacial adhesion strength to release the ink onto the receiver. Reproduced with permission from [80]; Copyright 2014, IEEE.

Capable of switching between temporary and permanent shapes, shape memory polymer (SMP) provides an alternative material to the conventional PDMS stamps in transfer printing. SMP can memorize the temporary shape formed by various pre-loads and easily recover its permanent shape after being triggered by an external stimulus (e.g., heat, electricity, light, moisture, etc.) [76,77,78,79]. In a representative use, the SMP stamp with a permanent shape of the relief structure, such as the example discussed in Figure 7, can be programmed to have a temporary shape of the collapsed state (Figure 10) [80,81]. The temporary shape with a large contact area and high interfacial adhesion strength is used in the pickup step. Upon heating, the SMP stamp recovers to its permanent shape with a significantly reduced contact area and low interfacial adhesion strength, which is then used for printing. Such temperature controlled transfer printing with the use of the SMP stamp is studied by an analytical model and the results provide optimized surface relief structures [82]. Though effective and easy to operate, the use of the SMP stamp may involve high temperatures, resulting in damage to delicate functional micro-devices and substrates. Moreover, it is not easy to apply in industrial-scale manufacturing.

### 2.7. Laser-Assisted Transfer Printing

**Figure 11:**
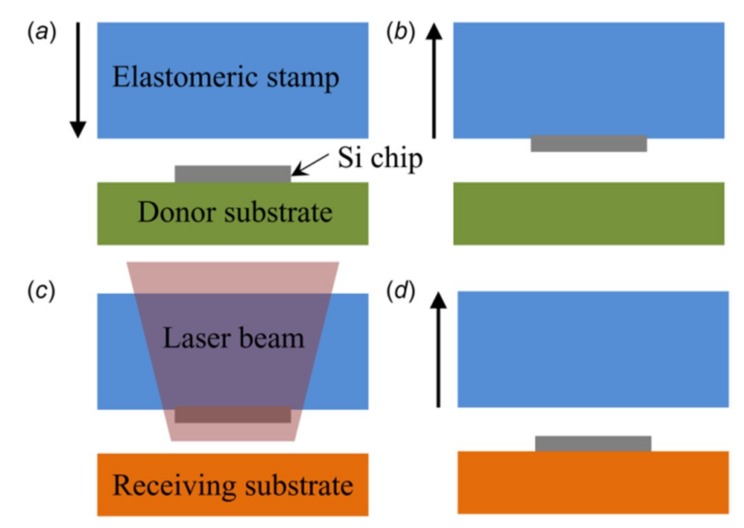
Schematic illustration of laser-assisted transfer printing. (**a**) Aligning the stamp with the donor substrate and (**b**) applying a rapid retraction on the stamp picks up the Si chip. (**c**) Applying a laser pulse initiates the separation at the device/stamp interface to (**d**) deliver the Si chip onto the surface of the receiver. Reproduced with permission from [88]; Copyright 2017, ASME.

Building on the idea of the thermal mismatch from the thermo-mechanical response of multilayered structures with different materials, a laser pulse is introduced in the printing step to initiate the separation of the device components from the stamp, where the device component does not need to be in contact with the receiver (Figure 11) [83,84]. The success of the non-contact laser-assisted transfer printing method closely depends on the laser pulse used in the printing step.

By avoiding direct contact between the functional device components and the target substrate, laser-assisted transfer printing expands the range of the receiving substrate (with different geometrical and material properties) in the integration process. To analyze the mechanism of delamination induced by the laser pulse, analytical mechanics models, validated by experiments and the finite element analysis, are also established to relate the energy release rate with the temperature distribution resulting from the laser pulse [33,85,86,87] and the influence of the device size on the temperature distribution is specifically analyzed [88]. Though it offers the versatility to print functional device components onto various flat, curved, or even partially defected substrate surfaces, laser-assisted transfer printing would require a relatively precise control of the intensity and irradiation time of the laser pulse, which is typically associated with expensive equipment.

### 2.8. Intaglio Transfer Printing Method

**Figure 12:**
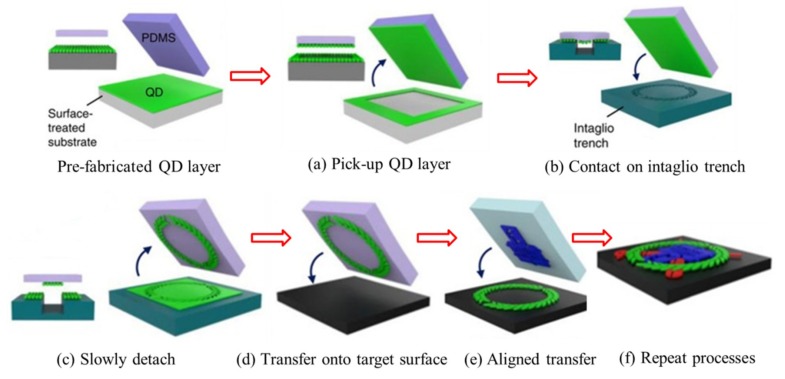
Schematic illustration of the transfer printing process of the pre-fabricated quantum dot (QD) layer: (**a**) The pre-fabricated QD layer is picked up quickly by the PDMS stamp; (**b**,**c**) the inked stamp is moved to contact on the intaglio trench and detached slowly to form QD patterns on the PDMS stamp; (**d**,**e**) the formed QD patterns are transfer printed on the target substrate; (**f**) QD arrays fabricated through repeat transfer printing processes. Reproduced with permission from [89]; Copyright 2015, Macmillan Publishers Limited.

Choi et al. [89] developed an intaglio transfer printing method for creating quantum dot (QD) arrays, which is slightly different from conventional methods. This method enabled the nanocrystal layers to be transfer-printed on various substrates without consideration of the size and shape. The whole transfer printing process is shown in Figure 12, in which the QD layer is firstly pre-fabricated on a Si source substrate. The flat PDMS stamp is in good contact with the QD layer to pick it up quickly. Then, the inked stamp is moved to contact on the intaglio trenches carved on the Si substrate (it is the special feature of this method) and is pressed with low pressure. In the third step, peeling off the stamp slowly makes the intaglio QD patterns form on the PDMS stamp rather than on the Si substrate, and the rest of the QD layer is released on the Si substrate. Finally, the inked stamp is transferred to print the QD layer onto the target substrate. Ultimate QD arrays can be completed through repetitive aligned transfer printing. It is worth mentioning that the current method can be generalized to transfer various QD layers regardless of the QD materials. Furthermore, the intaglio trenches are reusable because the residual QD layers on the trench can be eliminated easily through either mechanical rubbing or cleaning in a piranha solution.

### 2.9. Magnetic-Assisted Transfer Printing

**Figure 13:**
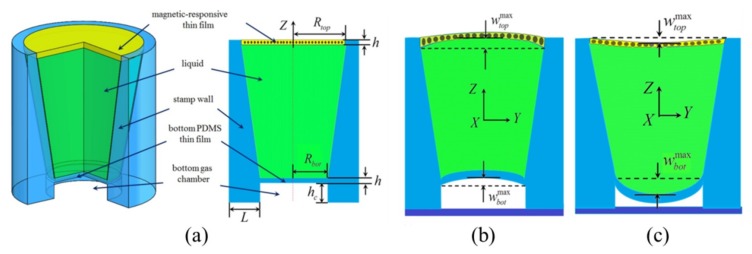
Schematic illustration of a structurally designed stamp consisting of an incompressible liquid chamber on top of a gas chamber in magnetic-assisted transfer printing. (**a**) 3D representation and cross-sectional views of the stamp design, with cross-sectional views of the stamp deformed upon the external magnetic actuation in the (**b**) pickup and (**c**) printing step. Reproduced with permission from [91]; Copyright 2018, ASME.

As an alternative approach to trigger the transfer printing by an external field, magnetic-assisted transfer printing was developed with the use of a magnetic-responsive film [90,91] or magnetic particles [92] to deform the elastic film. The former exploits a structurally designed stamp with an incompressible liquid chamber stacked on top of a gas chamber. The liquid chamber uses a magnetic-responsive thin film and a PDMS thin film at the top and bottom surface, respectively. In the pickup (printing) step, the top magnetic-responsive thin film is actuated by the external magnetic field to deform upward (downward), which transfers the motion to the bottom PDMS film and then induces a decreased (increased) pressure in the gas chamber (Figure 13). The pressure change in the gas chamber modulates the interfacial adhesion. The pressure change in the gas chamber as a function of the displacement profile of the top magnetic-responsive film is established in the analytical model, providing a comprehensive design guideline for the magnetic-assisted transfer printing.

With the above description of the various transfer printing methods, it can be safely concluded that each transfer method is associated with different characteristics for integration of the functional thin film devices on a compliant substrate. To help highlight the differences among the different transfer printing methods, we summarized the working principles, advantages, and limitations for each transfer printing method in Table 1.

## 3. Development and Applications of Transfer Printing in Flexible Electronics

**Figure 14:**
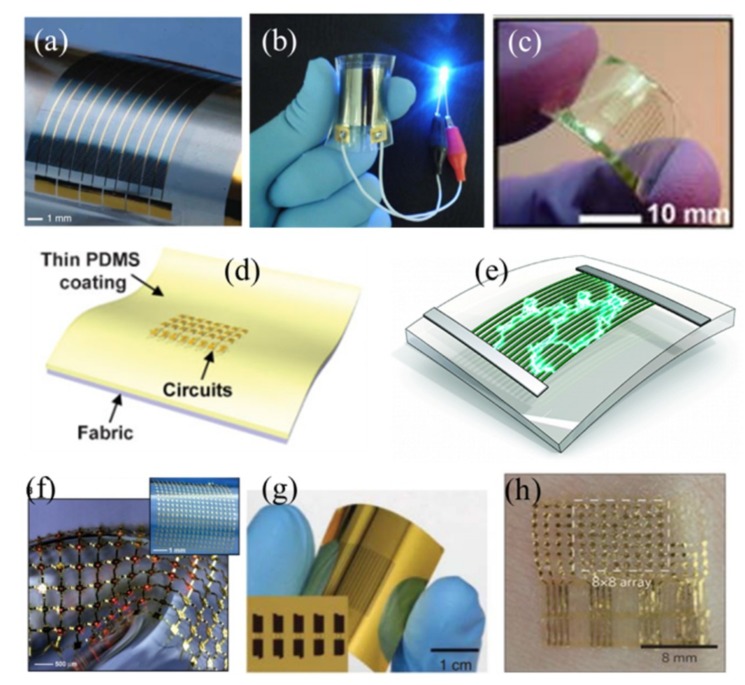
Various flexible and stretchable electronic devices integrated by the technique of transfer printing: (**a**) Flexible solar cell (Reproduced with permission from [140,141]; Copyright 2008, Nature Publishing Group); (**b**) a bendable rechargeable lithium-ion battery turning on a blue light-emitting diode upon bending (Reproduced with permission from [110]; Copyright 2012, American Chemical Society); (**c**) a stretchable thin film transistor on an elastomeric substrate (Reproduced with permission from [52]; Copyright 2005, American Institute of Physics); (**d**) a folded circuit integrated on a fabric substrate coated with a thin layer of PDMS (Reproduced with permission from [127]; Copyright 2009, WILEY-VCH Verlag GmbH & Co., KGaA, Weinheim); (**e**) a flexible piezoelectric energy conversion device (Reproduced with permission from [103]; Copyright 2010, American Chemical Society); (**f**) a stretchable inorganic LED on a rubber substrate (Reproduced with permission from [27]; Copyright 2009, American Association for the Advancement of Science); (**g**) a flexible capacitor (Reproduced with permission from [6,109]; Copyright 2010, IEEE); (**h**) a flexible temperature sensor array mounted on the skin (Reproduced with permission from [116]; Copyright 2013, Macmillan Publishers Limited).

Capable of assembling high-performance electronic devices onto a non-conventional (e.g., flexible or stretchable) substrate, the transfer printing technique has enabled wide-ranging applications from functional components to integrated device systems (Figure 14). The demonstrated functional components include transistors [22,23,35,41,52,93,94,95,96,97,98,99,100,101,102], energy-harvesting devices [103,104], light-emitting diodes (LED) [27,105,106,107,108], flexible capacitors [6,109], thin-film solar cells [110,111,112], memories [1,113,114], and various functional sensors [115,116,117,118,119,120,121,122,123]. A few representative system demonstrations are printed flexible integrated circuits [124,125,126,127,128,129], transient electronics [130,131,132,133,134,135,136,137], flexible displays [138,139], and many others.

### 3.1. Flexible Sensors

Various functional flexible sensors have been explored in bio-integrated electronics for the continuous monitoring of body temperature, pulse detection, emotion recognition, and exercise monitoring [142]. The application of transfer printing also goes beyond 2D sensors to heterogeneous 3D electronics [35], with applications in microfluidic devices, biological and chemical sensor systems, photonic and optoelectronic systems, and many others.

**Figure 15:**
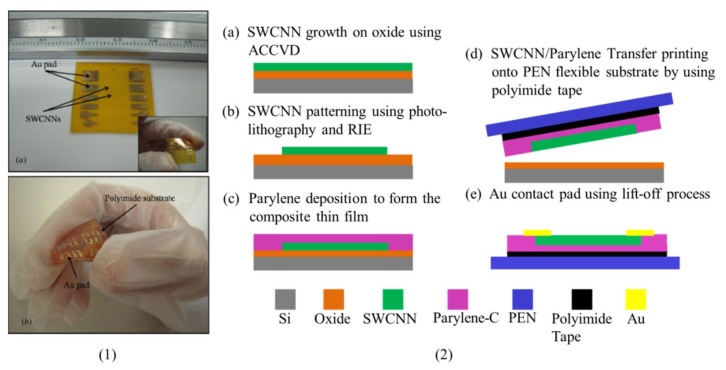
(1) Flexible strain-sensing devices fabricated on (**a**) a PEN and (**b**) PI substrate through transfer printing, with the (2) fabricating process of the single-walled carbon nanonet specimens. Reproduced with permission from [122]; Copyright 2011, IOP Publishing Ltd.

Transfer printing allows the assembly of nano-wires/micro-wires of GaAs, InP [21], and single-walled carbon nanotubes [22] into ordered arrays on plastic substrates. Combined with the technique of layer-by-layer assembly, contact transfer printing could use nanowires as building blocks to yield 3D multifunctional flexible electronics [143]. Due to the high surface-to-volume ratio, transferring hundreds of pre-aligned silicon nanowires onto plastic substrates results in ultrasensitive flexible chemical sensors [115]. A simple and fast nano-transfer printing (nTP) method that operates in ambient conditions [144,145] has shown potential to fabricate complementary inverter circuits, high-performance plastic transistors, and contact electrodes, which have electrical properties that are comparable to those fabricated with the conventional method [146]. Exploring the nTP method easily transfers the single-walled carbon nanonets (SWCNNs)-based flexible strain sensors that are fabricated on the Si substrate onto polyethylene naphthalate (PEN) and polyimide (PI) substrates with a superior strain sensitivity and linearity (Figure 15) [122].

**Figure 16:**
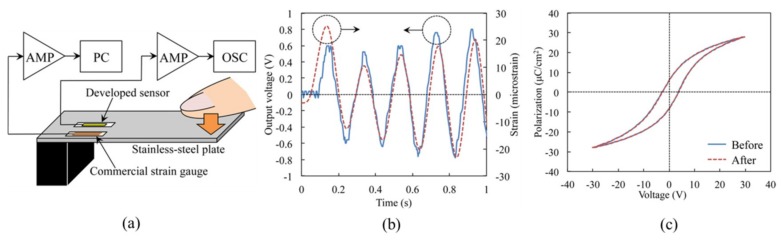
(**a**) Schematic illustration of the measurement setup with the fabricated sensor placed next to a commercial strain gauge on the surface of a stainless-steel plate. (**b**) Comparison of the voltage output from the fabricated sensor with the strain from the commercial strain gauge. (**c**) Almost identical polarization–voltage hysteresis curves of the piezoelectric strain sensors obtained before and after transfer printing demonstrate the effectiveness and applicability of the technique. Reproduced with permission from [118]; Copyright 2015, the Japan Society of Applied Physics.

With the use of an adhesive rubber stamp, the ultrathin piezoelectric strain sensor mounted onto an elastomeric substrate could measure the strain based on the voltage output with a resolution close to 10^−6^ (Figure 16) [118]. The magnetic sensor on the soft membrane can also be fabricated by a single-step direct transfer printing method [121].

### 3.2. Flexible Transistors

**Figure 17:**
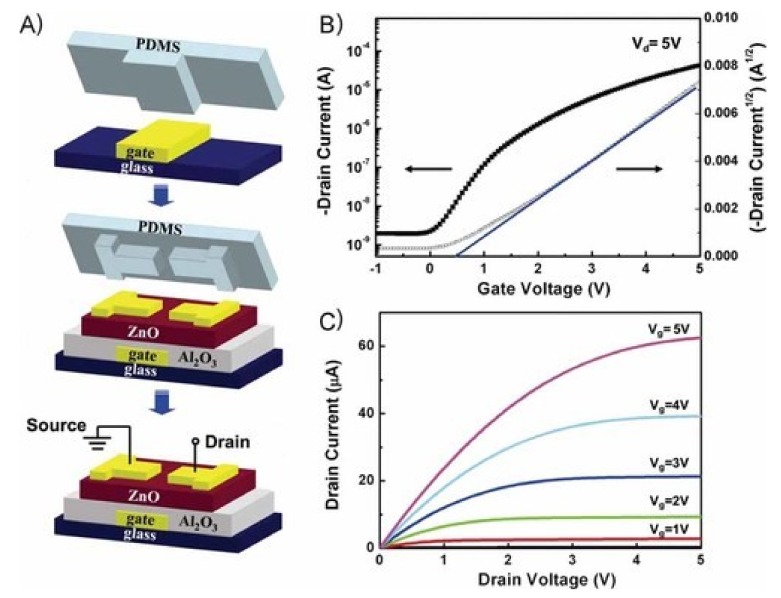
(**a**) Schematic illustration of the process of the fabrication of a ZnO-based thin film transistor with the water-mediated transfer printing. Device characterization of the fabricated thin film transistor: (**b**) Drain current-gate voltage transfer curves and (**c**) drain current-drain voltage output curves. Reproduced with permission from [64]; Copyright 2009, WILEY-VCH Verlag GmbH & Co., KGaA, Weinheim.

As an indispensable component in active devices, the transistor has been widely used in flexible sensors, displays, medical, and other electronic systems [147]. Transfer printing of single-crystal Si thin film transistors and GaAs microwires onto thin plastic sheets leads to flexible and bendable transistors [52,148] and metal semiconductor field-effect transistors (MESFETs) [147] that are robust to bending deformations. Similarly, transferring high electron mobility transistors to the plastic substrate yields the transistors in a bendable format that is robust to cyclic bending deformations [99]. The initial exploration of the single-crystal Si for stretchable transistors [12,24,149] was started with the creation of wavy Si thin ribbons on elastomeric substrates with the use of the pre-strain strategy. Exploring the technique of water-mediated transfer printing [63,64] also yields a ZnO-based thin film transistor with a high field-effect mobility (Figure 17).

**Figure 18:**
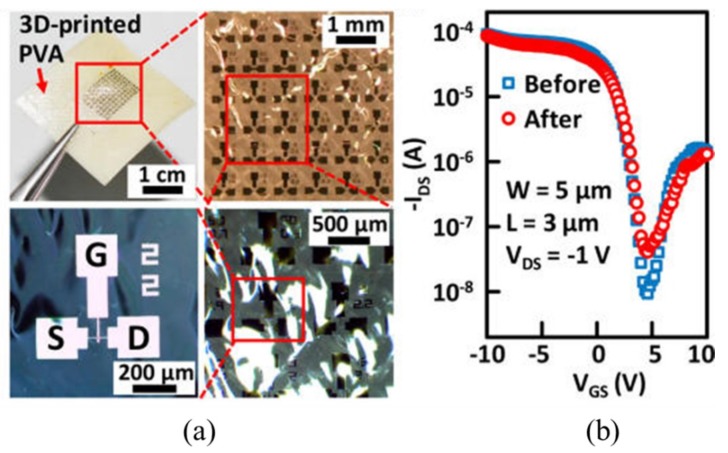
(**a**) Optical images of the fabricated carbon nanotube transistors via the water-assisted transfer printing and (**b**) transfer curves of the drain current versus gate voltage before and after transfer printing. Reproduced with permission from [130]; Copyright 2018, American Chemical Society.

The water-assisted transfer printing method [59] can also be used to transfer the pre-fabricated carbon nanotube (CNT)-based field effect transistors onto the surface of the 3D-printed water-soluble poly(vinyl alcohol) (PVA) substrate (Figure 18) [130]. The almost identical drain current versus gate voltage curves before and after transfer printing demonstrate the high fidelity of the method for fabricating high-performance CNT transistors.

Building on the contact transfer printing [143], the use of an octane and mineral oil mixture serves as a lubricant to help minimize the friction to result in highly ordered and aligned nanowires arrays for diodes and field-effect transistors [150]. Combined with a glass or polyethylene terephthalate (PET) substrate, highly aligned single-walled carbon nanotubes transferred at low temperatures yields a fully transparent thin film transistor [151]. Similarly, transparent field-effect transistors with high optical transmittance can be fabricated by transferring graphene thin films onto elastomeric substrates [152,153].

### 3.3. Solar Cells

**Figure 19:**
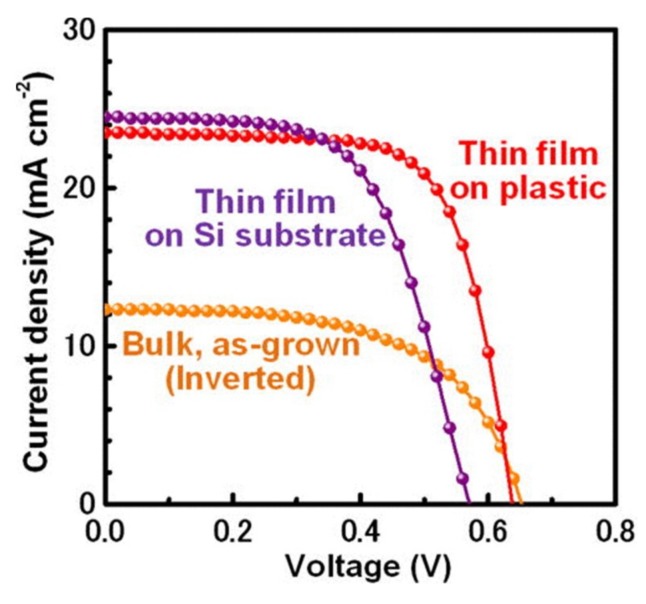
Current density as a function of the voltage of the highest-efficiency cells for each of the bulk reference cells and the thin-film cells on plastic and Si substrates, respectively. Reproduced with permission from [154]; Copyright 2012, American Institute of Physics.

Solar modules consisting of arrays of solar microcells that are fabricated on Si wafers can be integrated into diverse spatial layouts on flexible substrates using transfer printing on a large scale [140]. Comparison of the open-circuit voltages of the devices between those prior and after transfer printing (Figure 19) [154] demonstrates a high degree of power conversion efficiency [6,140].

**Figure 20:**
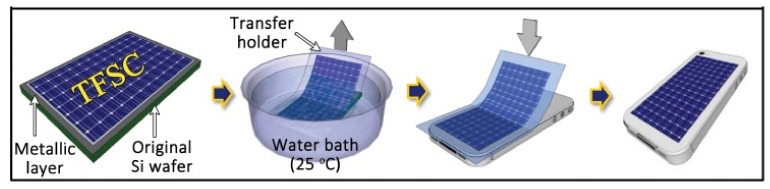
Schematic illustration of the peel-and-stick process for the fabrication of thin-film solar cells (TFSCs). Reproduced with permission from [112]; Copyright 2012, Macmillan Publishers Limited.

**Figure 21:**
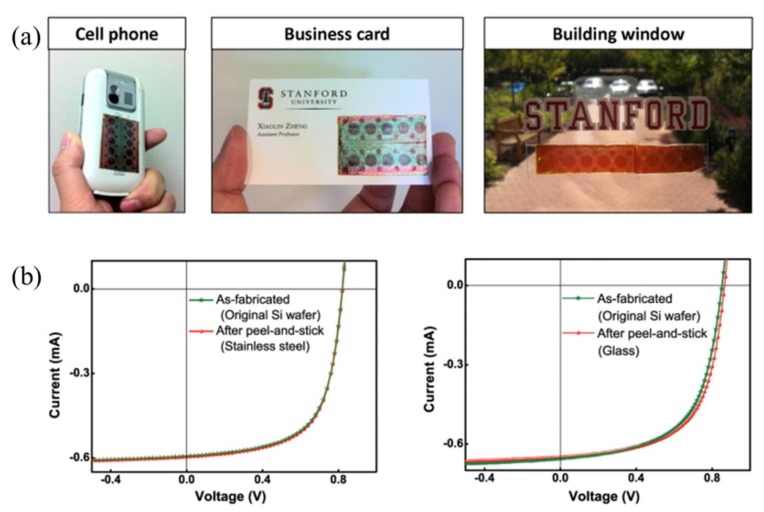
(**a**) TFSCs fabricated on a cell phone, business card, and building window, respectively; (**b**) comparison of the electrical performances of the TFSCs before and after the peel-and-stick process. Reproduced with permission from [112]; Copyright 2012, Macmillan Publishers Limited.

Building on the WTP method, a peel-and-stick process was developed to build thin-film solar cells (TFSCs) (Figure 20) on a nonconventional substrate, such as the surface of a cell phone, business card, and building window, without compromising its efficiency (Figure 21) [112,155].

### 3.4. Flexible Displays

Though the display is a core component of the human-computer interface, the conventional displays remain bulky and rigid. As an emerging technology, flexible displays are light and soft. Currently being explored as one of the key technologies in manufacturing flexible displays, transfer printing enables the transfer of various functional parts, such as the emissive or transport layers, from the growth substrate (donor) onto the device stack [138]. One representative example is the use of transfer printing for the integration of small crystalline-silicon circuits (chiplets) in the active-matrix organic light-emitting-diode (OLED) displays [139,156]. Prepared by the conventional photolithography, hundreds and thousands of chiplets could be transfer printed on the glass substrate via an elastomeric stamp to help build the integrated circuits. The OLED display is then formed over, and connected to, the chiplets. The resulting flexible OLED displays demonstrate an outstanding performance, highlighting the effectiveness of the transfer printing method for the fabrication of flexible displays.

**Figure 22:**
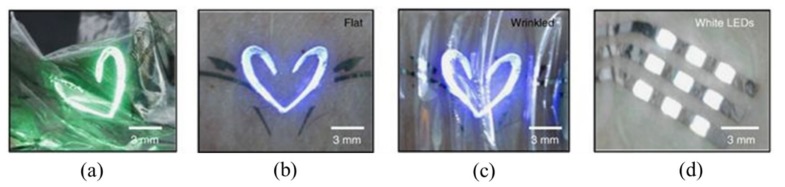
(**a**) Optical image of ultra-thin green QLEDs laminated on crumpled Al foil. Photographs of the blue QLEDs laminated on the human skin (**b**) before and (**c**) after deformation. (**d**) Optical image of white QLED arrays laminated on the human skin. Reproduced with permission from [89]; Copyright 2015, Macmillan Publishers Limited.

With the help of the intaglio transfer printing method discussed in Section 2.8, wearable red-green-blue and white quantum light-emitting diodes (QLEDs) with a high resolution were demonstrated [89]. The high electroluminescence efficiency in these QLEDs was maintained even after being laminated on crumpled Al foil or on human skin (Figure 22). After multiple printing processes, the red–green–blue diode array was aligned to yield a high-resolution display (ranging from 441 p.p.i. to 2460 p.p.i.). The successful demonstrations clearly confirm the applicability of the intaglio transfer printing method to fabricate ultra-high resolution full-color deformable QLED arrays.

### 3.5. Flexible Energy-Harvesting/conversion devices

Because of the intrinsically brittle nature of piezoelectric inorganic materials, piezoelectric energy harvesting and conversion devices fabricated on the conventional substrate are rigid. To exploit the bending property of the thin film device, the highly crystalline piezoelectric ribbons with nanometer scale thicknesses and micrometer-scale widths were transferred from the host substrate onto the pre-strained rubber substrates. Release of the pre-strain yields a stretchable energy harvesting device with a wavy/buckled structure that can be repeatedly stretched and released for multiple cycles without damage (e.g., crack) [103]. Additionally, the piezoelectric ribbons grown on a crystal substrate that were transferred onto the elastomeric substrate over a large area yields energy conversion devices with a high piezoelectric coefficient [104]. The transfer printing method was also explored to manufacture high-performance Pb(Zr,Ti)O_3_ (PZT) capacitors on plastic substrates for energy storage [109]. After forming the PZT films on the conventional wafer with the sol-gel method, an elastomeric stamp was used to transfer the thin films from the wafer onto the target plastic substrate. The front and backward bending tests (without damage) demonstrated that the flexible PZT thin film capacitor maintained its good electrical properties even under mechanical strain.

### 3.6. Flexible Electrodes

**Figure 23:**
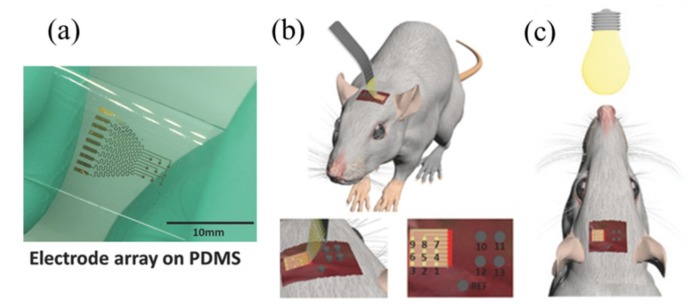
(**a**) A stretchable neural electrode array on the PDMS substrate is attached on the curvilinear surface of the brain in the rat to measure the (**b**) ECoG and (**c**) SSVEP signal, respectively. Reproduced with permission from [58]; Copyright 2017 by WILEY-VCH Verlag GmbH & Co., KGaA, Weinheim.

Using the thermal release transfer printing method, a stretchable neural electrode array with nine channels on a PDMS substrate was fabricated (Figure 23a) [58]. Attaching the device onto the dura mater of a rat in vivo allows the collection of electrocorticography (ECoG) signals (Figure 23b) and the detection of the steady-state visual evoked potentials (SSVEP) response induced by a flash (Figure 23c). Compared to a stainless-steel screw electrode consisting of four channels, the stretchable neural electrode array collected ECoG and SSVEP signals with higher signal-to-noise ratios, because of the conformal contact between the stretchable array and the curvilinear surface of the brain.

### 3.7. Other Devices

**Figure 24:**
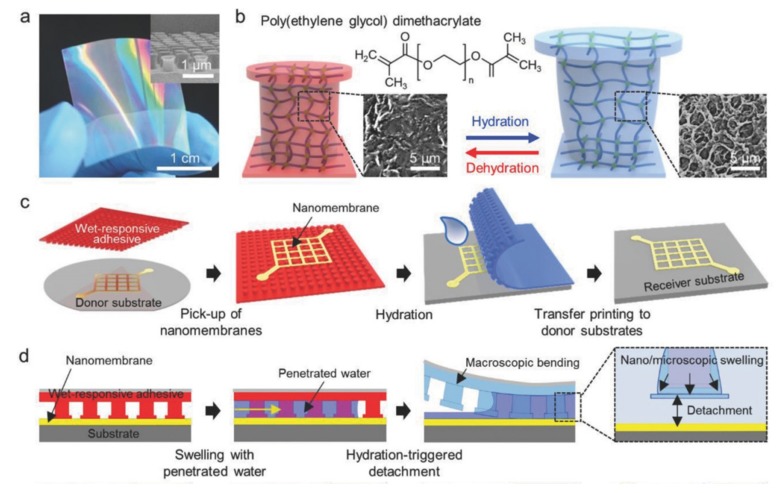
Schematic illustration of transfer printing with the PEGDMA hydrogel adhesive: (**a**) Photograph of the fabricated adhesive with a nanostructure array (the inset shows the SEM image); (**b**) the initial and expanded states of the nanostructures after hydration and dehydration; (**c**,**d**) schematic illustration of the transfer printing process using the smart hydrogel adhesive. Reproduced with permission from [40]; Copyright 2018, John Wiley & Sons, Inc.

**Figure 25:**
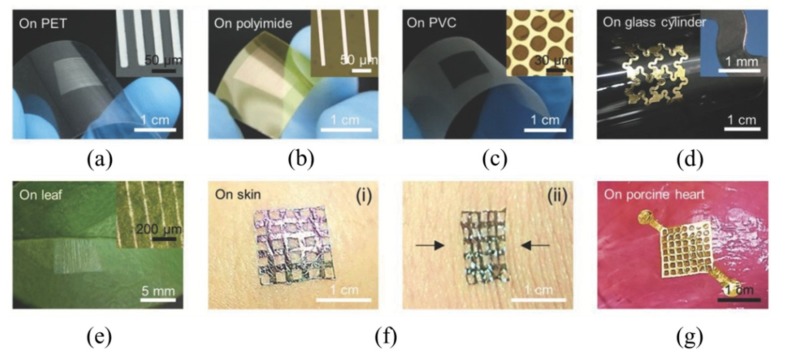
Different thin film devices transfer printed onto the surfaces of various substrates: (**a**) Ag nanoribbons, (**b**) Cu nanoribbons, and (**c**) Si micro-membranes transfer printed onto the PET, polyimide, and polyvinyl chloride (PVC) films, respectively; (**d**) the zig-zag shaped Au-polyurethane acrylate (PUA) composite membranes transfer printed onto the curved surface of a glass cylinder; (**e**) Ag nanoribbons transfer printed onto a plant leaf; (**f**) Pt-SU-8 composite membranes transfer printed onto human skin and attached firmly under the action of (i) tension and (ii) compression; (**g**) Au-SU-8 composite membranes transfer printed onto the porcine heart. Reproduced with permission from [40]; Copyright 2018, John Wiley & Sons, Inc.

Transfer printing is also explored to fabricate high-performance, stretchable integrated circuits toward personal health monitors and other biomedical devices [125]. Creating a cylindrical nanostructure array on a responsive hydrogel, such as poly(ethylene glycol) dimethacrylate (PEGDMA), provides a new transfer printing strategy [40]. When the hydrogel adhesive film is exposed to water, a significant volume expansion in the PEGDMA structure leads to a large increase in the diameter and height of the hydrogel arrays (Figure 24b). Because of the macroscopic bending in the hydrogel film induced by hydration, the strong surface adhesion strength (≈191 kPa) in the dry state of the hydrogel adhesive sharply decreases (≈0.3 kPa) upon water exposure. Because of the strong adhesion, bringing the hydrogel adhesive into contact with the donor substrate allows the retrieval of the nanomembranes (Figure 24c). After contacting the attached nanomembranes with the receiving substrate, the water dropped on one side penetrates into the space between the hydrogel adhesive and the receiving substrate, which induces the hydrogel to swell (Figure 24d). The swelling-induced bending deformation greatly reduces the adhesion strength at the adhesive/receiver interface, thereby releasing the nanomembranes onto the receiving substrate. The applicability of the smart PEGDMA adhesive for transfer printing was demonstrated by transferring various semiconducting and metallic (e.g., Si, Au, Ag, etc.) micro-/nano-membranes onto different receiving substrates (Figure 25).

## 4. Conclusions and Outlook

Transfer printing technology facilitates the heterogeneous assembly of different materials (e.g., micro/nano-materials) into various functional devices arranged in two-dimensional and three-dimensional layouts. This review describes the recent progress of transfer printing methods involved in the fabrication process of flexible and stretchable electronic devices. Several representative transfer printing methods and their working principles were briefly discussed. With this versatile technology, various materials (e.g., graphene thin films, nano-wires/micro-wires, single-crystal Si ribbons, etc.) and prefabricated device components can be effectively transferred from donor substrates onto receiving substrates with excellent electrical performance. Because each transfer printing method is associated with unique characteristics, careful selection of the transfer printing method for a specific application needs to be conducted, especially in the burgeoning field of flexible and stretchable electronics. Additionally, the optimization of the existing transfer printing methods and development of new methods would motivate continued and expanded efforts in the future.

Among the wide-ranging opportunities for transfer printing technology, one focus may lie in the design and optimization of active, programmable elastomeric stamps for efficient assembly of structures/devices with complicated patterns. Low cost, high speed, high resolution, and reliable transfer printing methods represent other promising areas for future research. The critical geometrical and material parameters that modulate the interfacial adhesion in each method also need to be extensively studied to provide physical insights. The strong interest of small and large companies also requires close examination of the feasibility and applicability of various transfer printing methods in fabricating electronic devices on an industrial scale. A promising route is to explore the roll-to-roll transfer printing process. Major innovations in machine design, process recipes, material handling, and system integration are urgently needed for the achievement of an efficient roll-to-roll transfer printing process. On the other hand, it is highly desirable to explore transfer printing techniques for the integration of functional devices onto curvilinear substrates with various material compositions or even biological materials, which will certainly open up new opportunities in bio-integrated electronics and human-machine interfaces.

## Figures and Tables

**Table 1:** Comparison of various transfer printing methods.

Method	Working Principle	Advantage	Limitation
Kinetically controlled transfer printing	Modulated by peeling velocity	Simple	Not suitable for viscoelastic substrates
Thermal release transfer printing	Modulated by peeling velocity and temperature	Simple, large range of modulation	Possible thermal damage
Water-assisted transfer printing	Modulated by a water layer	Easy to operate	Relatively complex operation
Surface relief structure-assisted transfer printing	Modulated by the change in contact area	Simple, large range of modulation	Inefficient
Shear-assisted transfer printing	Modulated by shear strain	Simple, high efficiency	Not suitable for viscoelastic substrates
Transfer printing based on shape memory polymer	Modulated by the change in contact area from shape memory effect	Simple	Possible thermal damage, inefficient
Laser-assisted transfer printing	Modulated by the laser-induced thermal mismatch	Non-contact, large range of modulation	Expensive equipment
Intaglio transfer printing method	Modulated by stress concentration and interface contact	Repeatable	Relatively complex operation
Magnetic-assisted transfer printing	Modulated by the pressure difference	Repeatable, high efficiency	Expensive
